# Androstane-3β,5α,6β,17β-tetrol tri­hydrate

**DOI:** 10.1107/S1600536811021349

**Published:** 2011-06-11

**Authors:** L. C. R. Andrade, M. J. B. M. de Almeida, J. A. Paixão, J. F. S. Carvalho, M. L. Sá e Melo

**Affiliations:** a CEMDRX, Department of Physics, University of Coimbra, P-3004-516 Coimbra, Portugal; b Centre for Neuroscience and Cell Biology, University of Coimbra, P-3004-517 Coimbra, Portugal; c Faculty of Pharmacy, University of Coimbra, P-3000-548 Coimbra, Portugal

## Abstract

The title hydrated tetrol, C_19_H_32_O_4_·3H_2_O, was synthesized by stereoselective reduction of the compound 3β,5α,6β-trihy­droxy­androstan-17-one. All rings are fused *trans*. The organic mol­ecules are connected head-to-tail along the *c* axis *via* O—H⋯O hydrogen bonds. Layers of water mol­ecules in the *ab* plane inter­connect these chains. A quantum chemical *ab initio* Roothan Hartree–Fock calculation of the isolated mol­ecule gives values for the mol­ecular geometry close to experimentally determined ones, apart from the C—O bond lengths, whose calculated values are significantly smaller than the measured ones, probably a consequence of the involvement of the C—OH groups in the hydrogen-bonding network.

## Related literature

For the synthesis of the title compound, see: Carvalho, Silva, Moreira *et al.* (2010 ▶); Carvalho, Silva & Sá e Melo (2010 ▶); Luche *et al.* (1978 ▶). For related structures, see: Andrade *et al.* (2011 ▶). For puckering parameters, see: Cremer & Pople (1975 ▶). For asymmetry parameters, see: Duax & Norton (1975 ▶); Altona *et al.* (1968 ▶). For reference bond-length data, see: Allen *et al.* (1987 ▶). For the program *GAMESS* used to perform the quantum chemical calculations, see: Schmidt *et al.* (1993 ▶).
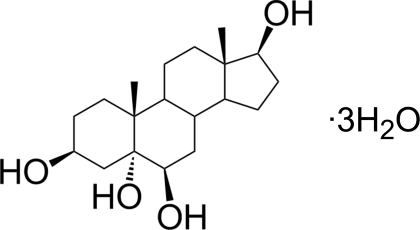


## Experimental

### 

#### Crystal data

C_19_H_32_O_4_·3H_2_O
*M* *_r_* = 378.49
Triclinic, 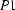

*a* = 5.8420 (2) Å
*b* = 7.3366 (2) Å
*c* = 12.7922 (3) Å
α = 74.560 (1)°
β = 83.091 (1)°
γ = 68.930 (1)°
*V* = 492.97 (2) Å^3^
*Z* = 1
Mo *K*α radiation
μ = 0.10 mm^−1^
*T* = 293 K
0.40 × 0.30 × 0.24 mm

#### Data collection

Bruker APEXII CCD area-detector diffractometer
Absorption correction: multi-scan (*SADABS*; Sheldrick, 2000 ▶) *T*_min_ = 0.973, *T*_max_ = 0.982
14489 measured reflections
2222 independent reflections
2132 reflections with *I* > 2σ(*I*)
*R*_int_ = 0.017

#### Refinement

*R*[*F* ^2^ > 2σ(*F* ^2^)] = 0.031
*wR*(*F* ^2^) = 0.082
*S* = 1.05
2222 reflections
259 parameters
9 restraints
H atoms treated by a mixture of independent and constrained refinement
Δρ_max_ = 0.20 e Å^−3^
Δρ_min_ = −0.18 e Å^−3^

### 

Data collection: *APEX2* (Bruker, 2006 ▶); cell refinement: *SAINT* (Bruker, 2006 ▶); data reduction: *SAINT*; program(s) used to solve structure: *SHELXS97* (Sheldrick, 2008 ▶); program(s) used to refine structure: *SHELXL97* (Sheldrick, 2008 ▶); molecular graphics: *PLATON* (Spek, 2009 ▶); software used to prepare material for publication: *SHELXL97*.

## Supplementary Material

Crystal structure: contains datablock(s) global, I. DOI: 10.1107/S1600536811021349/bt5554sup1.cif

Structure factors: contains datablock(s) I. DOI: 10.1107/S1600536811021349/bt5554Isup2.hkl

Additional supplementary materials:  crystallographic information; 3D view; checkCIF report

## Figures and Tables

**Table 1:** Hydrogen-bond geometry (Å, °)

*D*—H⋯*A*	*D*—H	H⋯*A*	*D*⋯*A*	*D*—H⋯*A*
O3—H3⋯O17^i^	0.82	1.98	2.787 (2)	169
O5—H5⋯O*W*1	0.82	2.08	2.891 (2)	170
O6—H6⋯O5^ii^	0.82	2.26	2.9897 (16)	149
O17—H17⋯O*W*3	0.82	1.94	2.718 (3)	159
O*W*1—H*W*11⋯O3^iii^	0.80 (2)	2.15 (2)	2.944 (2)	170 (4)
O*W*1—H*W*12⋯O*W*2^i^	0.82 (2)	2.19 (2)	2.977 (3)	160 (4)
O*W*2—H*W*21⋯O17	0.83 (2)	2.05 (2)	2.862 (2)	168 (4)
O*W*2—H*W*22⋯O3^iv^	0.81 (2)	2.14 (2)	2.921 (2)	161 (4)
O*W*3—H*W*31⋯O*W*1^v^	0.81 (2)	2.05 (2)	2.850 (3)	169 (5)
O*W*3—H*W*32⋯O*W*2^vi^	0.82 (2)	2.11 (2)	2.921 (3)	173 (5)

Symmetry codes: (i) 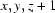; (ii) 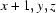; (iii) 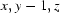; (iv) 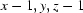; (v) 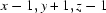; (vi) 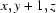.

**Fig. 1.**
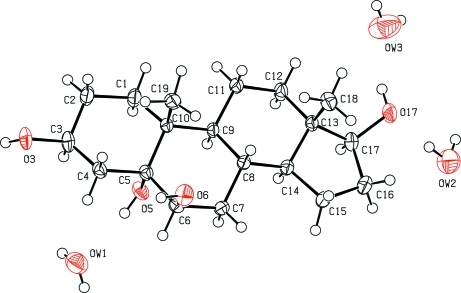
ORTEPII plot of the title compound. Displacement ellipsoids are drawn at the 50% level.

**Fig. 2.**
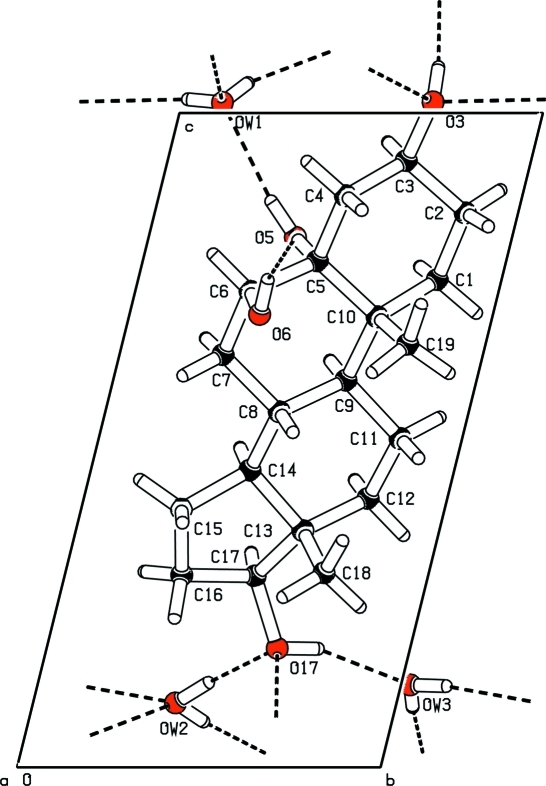
Projection of the crystal structure along the a axis, showing the H-bond network.

## supplementary crystallographic information

### Comment

Following our interest in oxysterols and their cytotoxicity (Carvalho, Silva, Moreira *et al.*, 2010), we were able to synthesize the title compound, (I), by stereoselective reduction of compound 3β,5α,6β-trihydroxyandrostan-17-one (Andrade *et al.*, 2011). Evaluation of the cytotoxicity of compound (I) towards HT-29 cancer cells (Carvalho, Silva, Moreira *et al.*, 2010) indicates no relevant values (IC_50_>50µ*M*), in contrast to other 3β,5α,6β-trihydroxy steroids, namely cholestane-3β,5α,6β-triol. Such result points to the importance of a C-17 cholesteryl-type side chain for cytoxicity. Determination of the three-dimensional structure of compound (I) by X-ray crystallography will contribute to correlate the importance of this side chain influence on the overall steroid geometry with such biological effect. Determined interatomic distances and valency angles agree well with expected values reported by Allen *et al.* (1987), except for C2–C3 bond [1.514 (3) Å] which is significantly shorter than average C*_sp_*^3^–C*_sp_*^3^ bond length [1.535 Å], a common feature with 3β,5α,6β-trihydroxyandrostan-17-one (Andrade *et al.*, 2011). Rings A, B and C have slightly flattened chair conformations [weighted average torsion angles 55.9 (8)°, 54.5 (4)°, 56.4 (9)°, respectively]. Ring D adopts a conformation in between 13β-envelope and 13β,14α-half chair [Cremer & Pople (1975) parameters q_2_ = 0.480 (2) Å and φ_2_ = 191.2 (3)°; asymmetry parameters (Duax & Norton, 1975; Altona *et al.*, 1968) ΔC_s_(14) = 24.64 (18)°; ΔC_s_(13) = 11.80 (19)°; ΔC_2_(13,14) = 9.1 (2)°; φ_m_ = 48.8 (1)°; Δ = 13.5 (3)°]. All rings are fused *trans*. The pseudo torsion angle C19—C10—C13—C18 is 2.86 (14)°, showing that the molecule is only slightly twisted.

There is an extensive hydrogen bonding network in the crystal sructure. The steroid molecules are linked head to tail *via* the O17 and O3 atoms, through a direct H bond where the O3 atom acts as a donor and through two additional H bonds mediated by a water molecule. The chains, aligned along the *c* axis, are further linked together *via* the two remaining water molecules. Interestingly the three water molecules are located in layers in the *ab* plane.

*Ab-initio* Roothan Hartree-Fock calculations of the free steroid molecule were performed using the computer code GAMESS (Schmidt *et al.*, 1993) in order to access the influence in the molecular geometry of the crystalline field, in particular of the solvent water molecules involved in H-bonding. These calculations gave values of the bond lengths,valency and torsion angles very close to those observed in the crystalline environment, except for the C–O bond lengths of the C—O—H groups, whose calculated values were significantely smaller than the measured ones, an effect that can be attributed to the influence of the hydrogen bonds (C17–O17 calc. 1.402, exp. 1.438 (2); C3–O3 calc. 1.408, exp. 1.4472 (18); C5–O5 calc. 1.423, exp. 1.4495 (19); C6–O6 calc. 1.405, exp. 1.426 (2) Å).

### Experimental

Synthesis of the title compound was performed using Luche conditions (NaBH_4_/CeCl_3_) (Luche *et al.*, 1978). Reduction of the carbonyl in position C17 revealed to be stereoselective rendering the 17β–OH in good yield (Carvalho, Silva & Sá e Melo, 2010). Crystallization from ethanol at room temperature afforded colourless crystals suitable for X-ray analysis. Analytical data of the title compound is in accordance with the literature (Carvalho, Silva & Sá e Melo, 2010). To a solution of 3β,5α,6β-trihydroxy-androstan-17-one (100 mg, 0.310 mmol) and CeCl_3_.7H_2_O (173.3 mg, 0.465 mmol) in THF (5 ml) and MeOH (5 ml) at 273 K, was slowly added NaBH_4_ (35.2 mg, 0.930 mmol). The mixture was stirred for 15 minutes, stopped with the addition of acetone, neutralized with Et_3_N and concentrated under vacuum. The residue was dissolved in ethyl acetate, filtrated and evaporated again. Flash chromatography (chloroform, ethanol 9:1) afforded the pure androstan-3β,5α,6β,17β-tetrol (I, 76.5 mg, 76%). *M*.p. 547 K (EtOH). IR (film) 3365, 2936, 2872, 1158, 1123, 1047, 1001, 960, 874, 745 cm^-1. 1^H NMR (300 MHz, DMSO–*d6*) δ p.p.m. 0.61 (3*H*, s, 18–CH_3_), 1.02 (3*H*, s, 19CH_3_), 1.85 (1*H*, dd, *J*=12.9, 11.2 Hz), 3.29 (1*H*, td, *J*=3.9, 3.3, 3.3 Hz, 6α–H), 3.43 (1*H*, dd, *J*=8.7, 4.5 Hz, 17α–H), 3.65 (1*H*, s, 5–OH), 3.79 (1*H*, tt, *J*=11.1, 5.7 Hz, 3α–H), 4.20 (1*H*, d, *J*=5.7 Hz, OH), 4.40 (1*H*, d, *J*=4.5 Hz, 17–OH), 4.41 (1*H*, d, *J*=3.3 Hz, 6–OH). ^13^C NMR (75 MHz, DMSO-*d6*δ p.p.m. 11.4, 16.3, 20.3 (CH_2_), 23.1 (CH_2_), 29.9 (CH_2_), 30.1, 31.1 (CH_2_), 32.0 (CH_2_), 34.1 (CH_2_), 36.8 (CH_2_), 37.9 (C), 40.9 (CH_2_), 42.6 (C), 44.8, 50.4, 65.7, 74.0, 74.3 (C–5), 80.1. MS m/*z* (%): 323.2 (28) [M–H]^+^, 311.6 (11), 294.1 (70), 281.5 (17), 266.3 (100), 263.7 (15), 98.8 (20).

### Refinement

All hydrogen atoms were refined as riding on their parent atoms using *SHELXL97* defaults except for those of the water molecules whose coordinates were refined from the starting coordinates obtained from a difference Fourier synthesis with *U*_eq_(H)=1.5*U*_eq_(O) using a *DFIX* restraint for the O—H bond of 0.82 Å and those of the C—OH groups which were positioned and refined with a SELXL97 HFIX 147 instruction.

The absolute configuration was not determined from the X-ray data, as the molecule lacks any strong anomalous scatterer atom at the Mo Kα wavelength, but was known from the synthetic route. Friedel pairs were merged before refinement.

### Crystal data

**Table:**

C_19_H_32_O_4_·3H_2_O	*Z* = 1
*M**_r_* = 378.49	*F*(000) = 208
Triclinic, *P*1	*D*_x_ = 1.275 Mg m^−^^3^
Hall symbol: P 1	Melting point: 547 K
*a* = 5.8420 (2) Å	Mo *K*α radiation, λ = 0.71073 Å
*b* = 7.3366 (2) Å	Cell parameters from 9795 reflections
*c* = 12.7922 (3) Å	θ = 3.1–27.9°
α = 74.560 (1)°	µ = 0.10 mm^−^^1^
β = 83.091 (1)°	*T* = 293 K
γ = 68.930 (1)°	Prism, colourless
*V* = 492.97 (2) Å^3^	0.40 × 0.30 × 0.24 mm

### Data collection

**Table:**

Bruker APEXII CCD area-detector diffractometer	2222 independent reflections
Radiation source: fine-focus sealed tube	2132 reflections with *I* > 2σ(*I*)
graphite	*R*_int_ = 0.017
φ and ω scans	θ_max_ = 27.9°, θ_min_ = 1.7°
Absorption correction: multi-scan (*SADABS*; Sheldrick, 2000)	*h* = −7→7
*T*_min_ = 0.973, *T*_max_ = 0.982	*k* = −9→9
14489 measured reflections	*l* = −16→16

### Refinement

**Table:**

Refinement on *F*^2^	Primary atom site location: structure-invariant direct methods
Least-squares matrix: full	Secondary atom site location: difference Fourier map
*R*[*F*^2^ > 2σ(*F*^2^)] = 0.031	Hydrogen site location: inferred from neighbouring sites
*wR*(*F*^2^) = 0.082	H atoms treated by a mixture of independent and constrained refinement
*S* = 1.05	*w* = 1/[σ^2^(*F*_o_^2^) + (0.0531*P*)^2^ + 0.0525*P*] where *P* = (*F*_o_^2^ + 2*F*_c_^2^)/3
2222 reflections	(Δ/σ)_max_ = 0.001
259 parameters	Δρ_max_ = 0.20 e Å^−^^3^
9 restraints	Δρ_min_ = −0.18 e Å^−^^3^

### Special details

**Table:**

Geometry. All e.s.d.'s (except the e.s.d. in the dihedral angle between two l.s. planes) are estimated using the full covariance matrix. The cell e.s.d.'s are taken into account individually in the estimation of e.s.d.'s in distances, angles and torsion angles; correlations between e.s.d.'s in cell parameters are only used when they are defined by crystal symmetry. An approximate (isotropic) treatment of cell e.s.d.'s is used for estimating e.s.d.'s involving l.s. planes.
Refinement. Refinement of *F*^2^ against ALL reflections. The weighted *R*-factor *wR* and goodness of fit *S* are based on *F*^2^, conventional *R*-factors *R* are based on *F*, with *F* set to zero for negative *F*^2^. The threshold expression of *F*^2^ > σ(*F*^2^) is used only for calculating *R*-factors(gt) *etc*. and is not relevant to the choice of reflections for refinement. *R*-factors based on *F*^2^ are statistically about twice as large as those based on *F*, and *R*- factors based on ALL data will be even larger.

### Fractional atomic coordinates and isotropic or equivalent isotropic displacement parameters (Å^2^)

**Table:**

	*x*	*y*	*z*	*U*_iso_*/*U*_eq_	
O3	0.6825 (3)	0.6903 (2)	1.01710 (10)	0.0341 (3)	
H3	0.5888	0.6824	1.0699	0.051*	
O5	0.5070 (2)	0.4040 (2)	0.81009 (10)	0.0274 (3)	
H5	0.5466	0.3118	0.8646	0.041*	
O6	1.1265 (2)	0.3581 (2)	0.69231 (12)	0.0327 (3)	
H6	1.1957	0.3562	0.7449	0.049*	
O17	0.3382 (3)	0.6345 (2)	0.18191 (10)	0.0322 (3)	
H17	0.2368	0.7464	0.1812	0.048*	
C8	0.7075 (3)	0.4785 (2)	0.54354 (13)	0.0190 (3)	
H8	0.8405	0.5298	0.5122	0.023*	
C9	0.5052 (3)	0.6405 (2)	0.59165 (13)	0.0184 (3)	
H9	0.3762	0.5836	0.6216	0.022*	
C10	0.5997 (3)	0.6831 (2)	0.68858 (13)	0.0189 (3)	
C4	0.7953 (3)	0.5133 (3)	0.87369 (14)	0.0235 (3)	
H4A	0.8595	0.3850	0.9256	0.028*	
H4B	0.9271	0.5678	0.8518	0.028*	
C5	0.7073 (3)	0.4805 (2)	0.77394 (13)	0.0200 (3)	
C11	0.3867 (3)	0.8294 (3)	0.50259 (14)	0.0265 (4)	
H11A	0.5074	0.8931	0.4723	0.032*	
H11B	0.2534	0.9239	0.5346	0.032*	
C13	0.4866 (3)	0.6246 (2)	0.36183 (13)	0.0203 (3)	
C14	0.5959 (3)	0.4377 (2)	0.45415 (13)	0.0209 (3)	
H14	0.4580	0.3953	0.4884	0.025*	
C1	0.3880 (3)	0.8233 (3)	0.74519 (14)	0.0283 (4)	
H1A	0.3224	0.9529	0.6945	0.034*	
H1B	0.2578	0.7667	0.7651	0.034*	
C3	0.5877 (3)	0.6566 (3)	0.92717 (14)	0.0285 (4)	
H3A	0.4634	0.5936	0.9554	0.034*	
C6	0.9057 (3)	0.3161 (2)	0.72770 (14)	0.0233 (3)	
H6A	0.9462	0.1899	0.7840	0.028*	
C7	0.8106 (3)	0.2850 (2)	0.63067 (14)	0.0240 (3)	
H7A	0.6831	0.2264	0.6555	0.029*	
H7B	0.9434	0.1898	0.5988	0.029*	
C2	0.4682 (4)	0.8545 (3)	0.84738 (15)	0.0326 (4)	
H2A	0.5831	0.9268	0.8266	0.039*	
H2B	0.3261	0.9360	0.8822	0.039*	
C12	0.2867 (3)	0.7820 (3)	0.41083 (14)	0.0267 (4)	
H12A	0.1527	0.7327	0.4392	0.032*	
H12B	0.2230	0.9044	0.3547	0.032*	
C18	0.6775 (3)	0.7118 (3)	0.29715 (15)	0.0293 (4)	
H18A	0.7901	0.6159	0.2601	0.044*	
H18B	0.7658	0.7405	0.3457	0.044*	
H18C	0.5963	0.8335	0.2450	0.044*	
C19	0.7914 (3)	0.7860 (3)	0.64653 (14)	0.0266 (4)	
H19A	0.7130	0.9184	0.6024	0.040*	
H19B	0.9156	0.7081	0.6040	0.040*	
H19C	0.8654	0.7961	0.7069	0.040*	
C17	0.3951 (3)	0.5244 (3)	0.29226 (14)	0.0262 (4)	
H17A	0.2486	0.4975	0.3281	0.031*	
C15	0.7529 (4)	0.2761 (3)	0.39465 (15)	0.0302 (4)	
H15A	0.7737	0.1420	0.4394	0.036*	
H15B	0.9131	0.2880	0.3739	0.036*	
C16	0.6017 (4)	0.3220 (3)	0.29381 (17)	0.0376 (5)	
H16A	0.7037	0.3314	0.2283	0.056*	
H16B	0.5340	0.2169	0.2990	0.056*	
OW1	0.6011 (4)	0.1107 (3)	1.01617 (15)	0.0493 (4)	
HW11	0.607 (7)	−0.002 (3)	1.021 (3)	0.074*	
HW12	0.468 (5)	0.163 (6)	1.045 (3)	0.074*	
OW2	0.1512 (3)	0.3921 (3)	0.09832 (16)	0.0519 (4)	
HW21	0.186 (7)	0.466 (5)	0.128 (3)	0.078*	
HW22	0.042 (6)	0.488 (5)	0.067 (3)	0.078*	
OW3	0.0382 (5)	1.0250 (4)	0.1259 (2)	0.0762 (7)	
HW31	−0.074 (7)	1.048 (8)	0.088 (4)	0.114*	
HW32	0.074 (9)	1.124 (6)	0.124 (4)	0.114*	

### Atomic displacement parameters (Å^2^)

**Table:**

	*U*^11^	*U*^22^	*U*^33^	*U*^12^	*U*^13^	*U*^23^
O3	0.0427 (8)	0.0482 (8)	0.0175 (6)	−0.0188 (7)	−0.0037 (5)	−0.0118 (6)
O5	0.0313 (7)	0.0332 (7)	0.0210 (6)	−0.0191 (6)	−0.0032 (5)	0.0002 (5)
O6	0.0222 (6)	0.0420 (8)	0.0344 (7)	−0.0078 (6)	−0.0039 (5)	−0.0129 (6)
O17	0.0371 (7)	0.0358 (7)	0.0209 (6)	−0.0056 (6)	−0.0087 (5)	−0.0087 (5)
C8	0.0199 (7)	0.0185 (8)	0.0183 (7)	−0.0047 (6)	−0.0020 (6)	−0.0058 (6)
C9	0.0200 (7)	0.0207 (8)	0.0148 (7)	−0.0058 (6)	−0.0022 (6)	−0.0055 (6)
C10	0.0209 (7)	0.0195 (8)	0.0160 (6)	−0.0053 (6)	−0.0032 (6)	−0.0047 (6)
C4	0.0259 (8)	0.0263 (9)	0.0183 (7)	−0.0089 (7)	−0.0057 (6)	−0.0033 (6)
C5	0.0220 (8)	0.0226 (8)	0.0174 (7)	−0.0103 (7)	−0.0022 (6)	−0.0037 (6)
C11	0.0323 (9)	0.0218 (8)	0.0200 (8)	0.0007 (7)	−0.0083 (7)	−0.0069 (7)
C13	0.0207 (7)	0.0232 (8)	0.0164 (7)	−0.0049 (6)	−0.0025 (6)	−0.0063 (6)
C14	0.0229 (8)	0.0214 (8)	0.0193 (7)	−0.0068 (7)	−0.0018 (6)	−0.0069 (6)
C1	0.0283 (9)	0.0310 (10)	0.0220 (8)	−0.0006 (8)	−0.0056 (7)	−0.0118 (7)
C3	0.0307 (9)	0.0420 (11)	0.0185 (8)	−0.0157 (8)	−0.0036 (7)	−0.0107 (7)
C6	0.0265 (9)	0.0190 (8)	0.0210 (7)	−0.0040 (7)	−0.0076 (6)	−0.0016 (6)
C7	0.0291 (9)	0.0182 (8)	0.0234 (8)	−0.0033 (7)	−0.0071 (6)	−0.0066 (6)
C2	0.0360 (10)	0.0354 (11)	0.0238 (8)	−0.0019 (8)	−0.0052 (8)	−0.0154 (8)
C12	0.0249 (8)	0.0302 (9)	0.0200 (8)	0.0007 (7)	−0.0065 (6)	−0.0092 (7)
C18	0.0314 (9)	0.0339 (10)	0.0243 (9)	−0.0149 (8)	−0.0006 (7)	−0.0050 (7)
C19	0.0348 (9)	0.0245 (9)	0.0245 (8)	−0.0157 (7)	−0.0044 (7)	−0.0033 (7)
C17	0.0288 (8)	0.0328 (10)	0.0198 (8)	−0.0113 (7)	−0.0026 (6)	−0.0089 (7)
C15	0.0393 (10)	0.0234 (9)	0.0251 (8)	−0.0024 (8)	−0.0064 (7)	−0.0104 (7)
C16	0.0535 (12)	0.0304 (10)	0.0286 (9)	−0.0064 (9)	−0.0088 (8)	−0.0145 (8)
OW1	0.0636 (11)	0.0451 (10)	0.0416 (9)	−0.0261 (9)	−0.0069 (8)	−0.0015 (7)
OW2	0.0525 (10)	0.0546 (11)	0.0552 (11)	−0.0203 (9)	−0.0148 (8)	−0.0151 (8)
OW3	0.0667 (14)	0.0515 (12)	0.107 (2)	−0.0064 (11)	−0.0284 (13)	−0.0217 (12)

### Geometric parameters (Å, °)

**Table:**

O3—C3	1.4473 (18)	C1—C2	1.538 (2)
O3—H3	0.8200	C1—H1A	0.9700
O5—C5	1.4496 (19)	C1—H1B	0.9700
O5—H5	0.8200	C3—C2	1.513 (3)
O6—C6	1.426 (2)	C3—H3A	0.9800
O6—H6	0.8200	C6—C7	1.520 (2)
O17—C17	1.438 (2)	C6—H6A	0.9800
O17—H17	0.8200	C7—H7A	0.9700
C8—C14	1.5248 (19)	C7—H7B	0.9700
C8—C7	1.525 (2)	C2—H2A	0.9700
C8—C9	1.547 (2)	C2—H2B	0.9700
C8—H8	0.9800	C12—H12A	0.9700
C9—C11	1.535 (2)	C12—H12B	0.9700
C9—C10	1.5603 (18)	C18—H18A	0.9600
C9—H9	0.9800	C18—H18B	0.9600
C10—C19	1.537 (2)	C18—H18C	0.9600
C10—C1	1.542 (2)	C19—H19A	0.9600
C10—C5	1.557 (2)	C19—H19B	0.9600
C4—C3	1.520 (3)	C19—H19C	0.9600
C4—C5	1.537 (2)	C17—C16	1.537 (3)
C4—H4A	0.9700	C17—H17A	0.9800
C4—H4B	0.9700	C15—C16	1.545 (2)
C5—C6	1.535 (2)	C15—H15A	0.9700
C11—C12	1.539 (2)	C15—H15B	0.9700
C11—H11A	0.9700	C16—H16A	0.9700
C11—H11B	0.9700	C16—H16B	0.9700
C13—C12	1.527 (2)	OW1—HW11	0.802 (19)
C13—C18	1.532 (2)	OW1—HW12	0.820 (19)
C13—C17	1.537 (2)	OW2—HW21	0.825 (19)
C13—C14	1.540 (2)	OW2—HW22	0.809 (19)
C14—C15	1.535 (2)	OW3—HW31	0.81 (2)
C14—H14	0.9800	OW3—HW32	0.82 (2)
			
C3—O3—H3	109.5	O3—C3—H3A	108.6
C5—O5—H5	109.5	C2—C3—H3A	108.6
C6—O6—H6	109.5	C4—C3—H3A	108.6
C17—O17—H17	109.5	O6—C6—C7	106.88 (14)
C14—C8—C7	110.14 (12)	O6—C6—C5	114.35 (13)
C14—C8—C9	108.20 (12)	C7—C6—C5	110.36 (13)
C7—C8—C9	111.03 (13)	O6—C6—H6A	108.4
C14—C8—H8	109.1	C7—C6—H6A	108.4
C7—C8—H8	109.1	C5—C6—H6A	108.4
C9—C8—H8	109.1	C6—C7—C8	113.45 (13)
C11—C9—C8	111.15 (13)	C6—C7—H7A	108.9
C11—C9—C10	114.18 (13)	C8—C7—H7A	108.9
C8—C9—C10	111.71 (12)	C6—C7—H7B	108.9
C11—C9—H9	106.4	C8—C7—H7B	108.9
C8—C9—H9	106.4	H7A—C7—H7B	107.7
C10—C9—H9	106.4	C3—C2—C1	111.83 (15)
C19—C10—C1	108.27 (14)	C3—C2—H2A	109.2
C19—C10—C5	111.98 (13)	C1—C2—H2A	109.2
C1—C10—C5	107.37 (13)	C3—C2—H2B	109.2
C19—C10—C9	109.47 (13)	C1—C2—H2B	109.2
C1—C10—C9	111.07 (13)	H2A—C2—H2B	107.9
C5—C10—C9	108.69 (12)	C13—C12—C11	111.20 (14)
C3—C4—C5	111.29 (14)	C13—C12—H12A	109.4
C3—C4—H4A	109.4	C11—C12—H12A	109.4
C5—C4—H4A	109.4	C13—C12—H12B	109.4
C3—C4—H4B	109.4	C11—C12—H12B	109.4
C5—C4—H4B	109.4	H12A—C12—H12B	108.0
H4A—C4—H4B	108.0	C13—C18—H18A	109.5
O5—C5—C6	105.43 (13)	C13—C18—H18B	109.5
O5—C5—C4	107.34 (13)	H18A—C18—H18B	109.5
C6—C5—C4	111.83 (13)	C13—C18—H18C	109.5
O5—C5—C10	106.41 (12)	H18A—C18—H18C	109.5
C6—C5—C10	114.03 (12)	H18B—C18—H18C	109.5
C4—C5—C10	111.26 (12)	C10—C19—H19A	109.5
C9—C11—C12	112.56 (14)	C10—C19—H19B	109.5
C9—C11—H11A	109.1	H19A—C19—H19B	109.5
C12—C11—H11A	109.1	C10—C19—H19C	109.5
C9—C11—H11B	109.1	H19A—C19—H19C	109.5
C12—C11—H11B	109.1	H19B—C19—H19C	109.5
H11A—C11—H11B	107.8	O17—C17—C16	109.69 (14)
C12—C13—C18	110.92 (15)	O17—C17—C13	116.41 (15)
C12—C13—C17	115.43 (13)	C16—C17—C13	105.03 (14)
C18—C13—C17	109.95 (14)	O17—C17—H17A	108.5
C12—C13—C14	108.13 (13)	C16—C17—H17A	108.5
C18—C13—C14	113.66 (13)	C13—C17—H17A	108.5
C17—C13—C14	98.26 (13)	C14—C15—C16	103.10 (15)
C8—C14—C15	119.76 (14)	C14—C15—H15A	111.1
C8—C14—C13	114.15 (12)	C16—C15—H15A	111.1
C15—C14—C13	103.82 (13)	C14—C15—H15B	111.1
C8—C14—H14	106.0	C16—C15—H15B	111.1
C15—C14—H14	106.0	H15A—C15—H15B	109.1
C13—C14—H14	106.0	C17—C16—C15	105.92 (14)
C2—C1—C10	112.90 (14)	C17—C16—H16A	110.6
C2—C1—H1A	109.0	C15—C16—H16A	110.6
C10—C1—H1A	109.0	C17—C16—H16B	110.6
C2—C1—H1B	109.0	C15—C16—H16B	110.6
C10—C1—H1B	109.0	H16A—C16—H16B	108.7
H1A—C1—H1B	107.8	HW11—OW1—HW12	104 (4)
O3—C3—C2	110.35 (15)	HW21—OW2—HW22	90 (4)
O3—C3—C4	109.14 (14)	HW31—OW3—HW32	114 (6)
C2—C3—C4	111.63 (15)		
			
C14—C8—C9—C11	−54.42 (17)	C19—C10—C1—C2	−64.85 (19)
C7—C8—C9—C11	−175.41 (13)	C5—C10—C1—C2	56.22 (19)
C14—C8—C9—C10	176.76 (13)	C9—C10—C1—C2	174.93 (15)
C7—C8—C9—C10	55.78 (17)	C5—C4—C3—O3	−177.17 (13)
C11—C9—C10—C19	−59.18 (19)	C5—C4—C3—C2	−54.92 (19)
C8—C9—C10—C19	68.02 (17)	O5—C5—C6—O6	−176.93 (13)
C11—C9—C10—C1	60.3 (2)	C4—C5—C6—O6	−60.60 (18)
C8—C9—C10—C1	−172.47 (13)	C10—C5—C6—O6	66.72 (16)
C11—C9—C10—C5	178.24 (14)	O5—C5—C6—C7	62.55 (17)
C8—C9—C10—C5	−54.57 (16)	C4—C5—C6—C7	178.88 (14)
C3—C4—C5—O5	−57.61 (18)	C10—C5—C6—C7	−53.80 (17)
C3—C4—C5—C6	−172.78 (14)	O6—C6—C7—C8	−71.50 (18)
C3—C4—C5—C10	58.43 (18)	C5—C6—C7—C8	53.39 (19)
C19—C10—C5—O5	177.61 (14)	C14—C8—C7—C6	−175.01 (14)
C1—C10—C5—O5	58.90 (15)	C9—C8—C7—C6	−55.17 (18)
C9—C10—C5—O5	−61.33 (15)	O3—C3—C2—C1	174.34 (15)
C19—C10—C5—C6	−66.61 (16)	C4—C3—C2—C1	52.8 (2)
C1—C10—C5—C6	174.68 (13)	C10—C1—C2—C3	−55.1 (2)
C9—C10—C5—C6	54.45 (16)	C18—C13—C12—C11	−69.96 (18)
C19—C10—C5—C4	61.00 (17)	C17—C13—C12—C11	164.15 (15)
C1—C10—C5—C4	−57.72 (16)	C14—C13—C12—C11	55.30 (19)
C9—C10—C5—C4	−177.94 (14)	C9—C11—C12—C13	−55.6 (2)
C8—C9—C11—C12	54.8 (2)	C12—C13—C17—O17	81.80 (19)
C10—C9—C11—C12	−177.68 (14)	C18—C13—C17—O17	−44.6 (2)
C7—C8—C14—C15	−56.0 (2)	C14—C13—C17—O17	−163.54 (14)
C9—C8—C14—C15	−177.54 (15)	C12—C13—C17—C16	−156.68 (16)
C7—C8—C14—C13	−179.93 (14)	C18—C13—C17—C16	76.94 (18)
C9—C8—C14—C13	58.54 (17)	C14—C13—C17—C16	−42.02 (17)
C12—C13—C14—C8	−59.23 (17)	C8—C14—C15—C16	−165.07 (15)
C18—C13—C14—C8	64.39 (18)	C13—C14—C15—C16	−36.31 (18)
C17—C13—C14—C8	−179.50 (13)	O17—C17—C16—C15	146.74 (16)
C12—C13—C14—C15	168.66 (14)	C13—C17—C16—C15	20.9 (2)
C18—C13—C14—C15	−67.72 (17)	C14—C15—C16—C17	9.3 (2)
C17—C13—C14—C15	48.38 (16)	C19—C10—C13—C18	2.85 (14)

### Hydrogen-bond geometry (Å, °)

**Table:**

*D*—H···*A*	*D*—H	H···*A*	*D*···*A*	*D*—H···*A*
O3—H3···O17^i^	0.82	1.98	2.787 (2)	169.
O5—H5···OW1	0.82	2.08	2.891 (2)	170.
O6—H6···O5^ii^	0.82	2.26	2.9897 (16)	149.
O17—H17···OW3	0.82	1.94	2.718 (3)	159.
OW1—HW11···O3^iii^	0.80 (2)	2.15 (2)	2.944 (2)	170 (4)
OW1—HW12···OW2^i^	0.82 (2)	2.19 (2)	2.977 (3)	160 (4)
OW2—HW21···O17	0.83 (2)	2.05 (2)	2.862 (2)	168 (4)
OW2—HW22···O3^iv^	0.81 (2)	2.14 (2)	2.921 (2)	161 (4)
OW3—HW31···OW1^v^	0.81 (2)	2.05 (2)	2.850 (3)	169 (5)
OW3—HW32···OW2^vi^	0.82 (2)	2.11 (2)	2.921 (3)	173 (5)

Symmetry codes: (i) *x*, *y*, *z*+1; (ii) *x*+1, *y*, *z*; (iii) *x*, *y*−1, *z*; (iv) *x*−1, *y*, *z*−1; (v) *x*−1, *y*+1, *z*−1; (vi) *x*, *y*+1, *z*.

## References

[bb1] Allen, F. H., Kennard, O., Watson, D. G., Brammer, L., Orpen, A. G. & Taylor, R. (1987). *J. Chem. Soc. Perkin Trans. 2*, pp. S1–19.

[bb2] Altona, C., Geise, H. J. & Romers, C. (1968). *Tetrahedron*, **24**, 13–32. 10.1016/s0040-4020(01)83329-9 6037289

[bb3] Andrade, L. C. R., Almeida, M. J. B. M. de, Paixão, J. A., Carvalho, J. F. S. & Sá e Melo, M. L. (2011). *Acta Cryst.* E**67**, o1056–o1057. 10.1107/S1600536811011706 PMC3089281 21754383

[bb4] Bruker (2006). *APEX2* and *SAINT* Bruker AXS Inc., Madison, Wisconsin, USA.

[bb5] Carvalho, J. F. S., Silva, M. M. C., Moreira, J. N., Simões, S. & Sá e Melo, M. L. (2010). *J. Med. Chem.* **53**, 7632–7638. 10.1021/jm1007769 20931970

[bb6] Carvalho, J. F. S., Silva, M. M. C. & Sá e Melo, M. L. (2010). *Tetrahedron*, **66**, 2455–2462.

[bb7] Cremer, D. & Pople, J. A. (1975). *J. Am. Chem. Soc.* **97**, 1354–1358.

[bb8] Duax, W. L. & Norton, D. A. (1975). *Atlas of Steroid Structure* New York: Plenum Press.

[bb9] Luche, J. L., Rodriguez-Hahn, L. & Crabbé, P. (1978). *J. Chem. Soc. Chem. Commun.* **14**, 601–602.

[bb10] Schmidt, M. W., Baldrige, K. K., Boatz, J. A., Elbert, S. T., Gordon, M. S., Jensen, J. J., Koseki, S., Matsunaga, N., Nguyen, K. A., Sue, S., Windus, T. L., Dupuis, M. & Montgomery, J. A. (1993). *J. Comput. Chem.* **14**, 1347–1363.

[bb11] Sheldrick, G. M. (2000). *SADABS* University of Göttingen, Germany.

[bb12] Sheldrick, G. M. (2008). *Acta Cryst.* A**64**, 112–122. 10.1107/S0108767307043930 18156677

[bb13] Spek, A. L. (2009). *Acta Cryst.* D**65**, 148–155. 10.1107/S090744490804362X PMC2631630 19171970

